# Are family practice trainers and their host practices any better? comparing practice trainers and non-trainers and their practices

**DOI:** 10.1186/1471-2296-14-23

**Published:** 2013-02-21

**Authors:** Pieter van den Hombergh, Saskia Schalk-Soekar, Anneke Kramer, Ben Bottema, Stephen Campbell, Jozé Braspenning

**Affiliations:** 1 IQ healthcare (114), Radboud University Nijmegen Medical Centre, PO Box 9101, 6500, HB, Nijmegen, the Netherlands; 2 Department of Psychology, Tilburg University, PO Box 90153, 5000, LE, Tilburg, the Netherlands; 3 Department of Primary and Community Care (117), Radboud University Nijmegen Medical Centre, PO Box 9101, 6500, HB, Nijmegen, the Netherlands; 4 Health Sciences Research Group – Primary Care, University of Manchester, Williamson Building, Oxford Road, Manchester, M13 9, PL, UK

**Keywords:** Primary care, Family practice, Quality of healthcare, Teaching, Workload

## Abstract

**Background:**

Family Physician (FP) trainees are expected to be provided with high quality training in well organized practice settings. This study examines differences between FP trainers and non-trainers and their practices to see whether there are differences in trainers and non-trainers and in how their practices are organized and their services are delivered.

**Method:**

203 practices (88 non-training and 115 training) with 512 FPs (335 non-trainers and 177 trainers) were assessed using the “*Visit Instrument Practice organization* (VIP)” on 369 items (142 FP-level; 227 Practice level). Analyses (ANOVA, ANCOVA) were conducted for each level by calculating differences between FP trainees and non-trainees and their host practices.

**Results:**

Trainers scored higher on all but one of the items, and significantly higher on 47 items, of which 13 remained significant after correcting for covariates. Training practices scored higher on all items and significantly higher on 61 items, of which 23 remained significant after correcting for covariates. Trainers (and training practices) provided more diagnostic and therapeutic services, made better use of team skills and scored higher on practice organization, chronic care services and quality management than non-training practices. Trainers reported more job satisfaction and commitment and less job stress than non-trainers.

**Discussion:**

There are positive differences between FP trainers and non-trainers in both the level and the quality of services provided by their host practices. Training institutions can use this information to promote the advantages of becoming a FP trainer and training practice as well as to improve the quality of training settings for FPs.

## Background

Family physician (FP) trainers and their host practices are expected to be places of excellence in order to provide a predetermined standard of medical education. Some evidence for this hypothesis is already available, which shows differences between FP trainers and non-trainers and their practices, although the data are mainly from the 1990s [1-4]. Three of these studies found FP trainers to be better qualified than non-trainers for some organizational competencies like equipment and delegation [1,3,4]. Many Colleges, such as the College of Family Physicians of Canada or the Royal College of General Practitioners in the UK, have a responsibility in setting the standards for the training, in certification and lifelong education of FPs. Vocational training has become compulsory in the European Union, requiring a high-standard of training and methods to assess the quality of the training [5,6]. FP training institutes are obliged to provide trainees with professional FP trainers working in excellent practice settings [7,8]. We need therefore information on the quality and added value of training practices and FP trainers [9]. Providing excellence in training requires more than the definition of standards for FP trainers and their practices alone [10]. In the Netherlands FP trainers receive instruction and training to become a teacher and clinical supervisor, [8] and training practices are stimulated to participate in a practice accreditation program providing detailed feedback to the FP and the practice (Appendix 1) [11-13]. The feedback offers trainers, training practices, and institutions detailed information that could help to show where improvement in organization is needed [14]. Moreover, it makes explicit what the added value and advantages are of being a trainer for both the training practice and the FP.

The aim of this study was to explore differences in the structure and process measures between FP trainers and non-trainers and their practices, to see whether there is added value in terms of the quality of services provided to patients and in the quality of the practice organization of training practices as a host organization for trainees.

## Methods

### Setting and design

335 FP non-trainers and 177 FP trainers voluntarily participated in the practice accreditation program in 2006–2007. A practice is denoted as a training practice when at least one FP trainer for postgraduate training is employed in that practice; a training practice can have therefore both FP trainers and non-trainers. There were 88 non-training practices (34.9% single-handed) with a total of 164 FPs, and 115 training practices (16.5% single-handed) with a total of 348 FPs. Sixty-two practices (30.5%) were practices with two FPs (32 non-training and 30 training practices). Seventy-six practices (37.4%) were group practices comprising 1 to 6 FP trainers, and 1 to 8 FP non-trainers. The practices were spread all over the Netherlands. All practices agreed on the use of the data at an aggregated level. Having this kind of informed consent a separate ethical approval is not required under Dutch law.

### Instrument and procedure

The Visitation Instrument to assess Practice organization [11] (VIP) was used to collect the data, which contains 369 items; 227 items at the practice level and 142 at the FP level. The VIP-tool includes all items of the international validated European Practice Assessment indicators (EPA) [15]. It uses a combination of questionnaires that are completed by FPs and staff members, patient questionnaires and observational checklists completed by trained independent observers. These trained observers collected and processes the data from the questionnaires and the observation in the practice in a database for analysis. For a full description of the method and the process of data collection we refer to a previous publication [11]. Over the years some items have been changed to adjust the instrument to the permanent changes in GP-care.

The questionnaires focused on infrastructure (premises and equipment, practice management), the team (delegation to staff, cooperation with other care providers, service and organization, administration, workload (hours per week), job stress (scales), information (record keeping, patient information) and quality management (CME, QI), see Table 1 and 2. All items were answered on a ‘yes’ or ‘no’ basis (except for workload and job stress that used Likert scales). We also collected data on FP and the practice characteristics, see Table 3.

**Table 1:** Differences between FP trainers and non-trainers in organization of care, (FP-level, n=512)

**Main categories & subcategories**		***N*. of items**	***N*. of items**^**Δ**^	Items that differed after covariate correction*	**Effect size Cohen’s *d #***
**I Infrastructure**					
	1. Surface of waiting room & FP’s office	2	1		
*Premises Medical equipment*	1. Medical equipment in the practice	17	5		
	2. Number of vials	3			
	3. Content of FP’s bag	19	3		
	4. Use of instruments/diagnostics	12	10	Hyfrecator	.27
				Electrocardiograph EKG	.22
				Sims-Hühner test	.19
				Audiometer	.23
				Doppler device	.27
				Peak flow meter	.22
	5. Applying technical skills	14	8	Examining fluor slide	.19
				Removing lipoma/ atheroma	.23
	6. Eye diagnostics	9	5	Lenses of −0.5 & +0.5 D	.27
				Stenopeic aperture	.24
**II Team**					
***Workload (hrs/wk)***	1. Activities directly patient-related	3	2	Consultations, visits & calls	.20
	2. Activities indirectly patient-related	8	3		
	3. Quality Improvement (+ CME)	3			
	4. Other professional activities (meetings)	1			
	5. Total of 1–4 = time/ week in practice	4			
	6. Workload/ week (all activities)	5	2		
	7. Breaks	2			
***Job stress (5 scales) 1 scale= 1 item***	1. Working with pleasure & commitment	4 (1)	1	Work w. pleasure & commitment	.24
	2. Being busy with irrelevant tasks	4 (1)			
	3. Satisfaction with available time for tasks	5 (1)			
	4. Satisfaction with investment on patients	3 (1)	1		
	5. Burnout at the end of the day	16 (1)			
**III Information**					
*Record keeping Patient info*	1. Quality of electronic medical records	4			
	2. Using FP Information System	11	2		
	1. Frequency of handing out patient info	1			
	2. Organizing patient information	10	1		
**IV Quality management**					
***Q Assessment***	1. Assessing/testing medical skills	9	3	Video record of consultation	1.57
**Total**	**Total number of items**	**142**	**47**	**13**	

**Δ***Number of items that differed significantly between the two groups*

* Covariates: gender, age, years of experience, weekly hours worked, and number of patients.

# Significant after covariate correction.

**Table 2:** Differences between FP training & non-training practices in organization of care (practice level, n=203)

**Categories & subcategories**	**Topics within subcategory**	***N*. of items**	***N*. of items**^**Δ**^	Items that differed after covariate* correction	**Effect size Cohen’s *d#***
**I Infrastructure**					
***Premises***	1. m [2] of waiting room and FP’s office	4	0		
***Medical equipment & Hygiene***	2. Office equipment	10	3		
	1. Hygiene	11	2		
	2. Emergency care	13	3	EKG	.33
	3. Special instruments/equipment	13	6	Audiometer	.47
				Hyfrecator	.32
	4. Availability of lab tests	8	5	Peak flow meter	.46
				Digital Hb meter	35
				ESR	35
				Occult blood in faeces	35
***Accessibility Services & organization***	1. Waiting time for answering telephone	1	0		
	1. Organization of the practice	17	3		
	2. Preventive service of the practice	1	1		
	3. Preventive tasks provided by practice	11	2		
**II Team**					
***Delegated tasks***	1. Medical-technical and diagnostic tasks	14	10	Nitrogen treatment	.33
				Compression therapy	.53
				Removing splinters	.41
				Vena puncture	.42
				Taping ankle sprain	.31
				Audiometry	.40
				Making EKGs	.40
***Collaboration with colleagues***	2. Chronic diseases & prevention tasks	15	1	Spirometry	.43
	3. Organization and administration	9	1		
	1. Time meeting with staff	2	0		
	2. Time meeting with colleagues	2	0		
	3. Collaboration in the group practice	11	4		
	4. Time meeting other prim. care providers	6	2		
	5. Collaborating with prim. care providers	7	0		
	6. Collaborating with the hospital	4	1		
	7. Consultations of specialist/ consultants	10	2		
	8. Collaborating with other care providers	11	0		
**III Information**					
***Record keeping***	1. Computerized Medical Records	9	0	Risk factors for CVD	.33
	2. Electronic communication	6	1		
	3. Use of separate prevention module	3	2		
***Patient info***	1. Supplying patient info by practice	6	1		
**IV Quality management**					
***FP group Practice***	1. Organization of quality in group practice	8	3		
	2. Quality policy within the practice	15	9	Calibration/maintenance	.26
				Patient survey	32
				No commercial leaflets	34
				Annual report	44
				Tel. advice by staff using	
				Dutch College guidelines	42
				Policy for CME of staff	32
				Appraisal for staff	.23
**Total**	**Total number of items**	**227**	**61**	**23**	

**Δ** Number of items that differed significantly between the two groups

*Covariates: type of practice, practice location, urbanization level, number of patients, fte nurse per 1,000 patients, fte management support per 1,000 patients, FP years working in current practice.

# significant after covariate correction.

**Table 3:** Characteristics of family physicians and their practices

**FP characteristics**	**Mean**	**Non-trainers**	**FP trainers**	**Cohen’s *d***
***N *= 512**	**& SD**	***N *= 335**	***N *=177**	*
Gender	*Men*	176	129	−0.42
	*Women*	157	47	
		*2 unknown*	*1 unknown*	
Age	M	44.05	49.95	0.77
	SD	8.31	6.27	
Years in practice	M	12.94	19.42	0.75
		9.13	7.70	
	SD	*1 unknown*	*2 unknown*	
Proportion of full-time FPs	M	0.69	0.79	0.53
		0.21	0.16	
	SD	*1 unknown*		
Total number of patients	M	1,795.6	2,069.76	0.40
		731.06	603.56	
	SD	*12 unknown*	*1 unknown*	
**Practice characteristics**	**Mean**	**Non-trainer**	**FP trainer**	Cohen’s d *
***N *= 203**	**& SD**	**practices**	**practices**	
		***N *= 88**	***N *= 115**	
Type of practice	M	2.62	3.08	0.29
1: single-handled	SD	1.7		
2: duo				
3: group				
Practice location	M	1.71	1.85	0.35
1: next to FP’s house	SD	0.45	0.36	
2: not next to FP’s house				
Urbanization level	M	2.33	2.67	0.34
1: rural < 5000, 2: village 5 -	SD	1.02	0.97	
30.000, 3; small town 30–100.00,				
4: large town > 100.000.				
Number of patients	M	3,970.75	5,553.82	0.57
	SD	2,530.87	2,969.41	
Fte practice nurse per 1,000 patients	M	0.41	0.44	0.68
	SD	0.10	0.10	
Fte management support /1,000 patients	M	0.02	0.04	0.30
	SD	0.03	0.11	
Years worked in current practice	M	12.09	14.14	0.33
	SD	6.82	5.29	

* A positive d means that the mean scores for FP trainers were higher than for non-trainers.

### Analyses

The differences between FP trainers and non-trainers were calculated for each of the 142 FP-level items with a one-way analysis of variance (ANOVA). Cohen’s *d* has been used to estimate effect sizes from the quantitative and dimensional measures. Because of the large number of multiple comparisons involved, the effect sizes (Cohen’s *d* ) were only calculated for the significant differences (p<0.05). Cohen suggested effect sizes in terms of: d=0.2 is small, 0.5 moderate and 0.8 large [16]. When the covariates (gender, age, years of practice experience, weekly hours worked, and number of patients) were significantly different for the two groups, an analysis of covariance was used (ANCOVA). We considered the significant differences between the two groups only for those items for which an effect size was calculated. We will present effect sizes only for those items that differed significantly (p<0.05) after the covariate analysis. The differences between the 227 practice level items for training and non-training practices were analyzed in the same way. The covariates to be corrected for were: type of practice (single handed, two FPs, more than two FPs, health care centre), practice location (next to FP’s house or not), urbanization level (small village, medium to large town, medium size city, or large city), number of patients, weekly hours worked (fte) per 1,000 patients for the nurse, weekly hours worked (fte) per 1,000 patients for management support, and the number of years the FP has worked in the current practice.

## Results

The characteristics of the participating FPs and practices are shown in Table 3. FP trainers were more often male, older, had more experience as a FP, worked longer hours, and had more patients. FP training practices were more often suburban practices with more partners, more patients, more assistance and more management support. The 177 FP trainers scored higher on all except one (UV-lamp for eye diagnostics) of the 142 FP-level items and significantly higher on 47 of the 142 items (Table 1). Training practices scored higher on all 227 practice level items and significantly higher on 61 of the 227 items (Table 2). Most of the differences were found in ‘medical equipment’. The differences between FP trainers and non-trainers that were significant after correction for covariates are described below in more detail for FP and practice level respectively.

### FP level

After correction for covariates, FP trainers reported carrying out diagnostic activities more often than non-trainers; such as audiometry, Doppler for detecting peripheral arterial obstruction, EKG, spirometry, Sims Hühner test for infertility and eye-diagnostics (using a stenopeic aperture, 0.5 D lenses for testing refraction testing, UV-penlight, fundoscopy) and lab (KOH microscopic examination of fluor vaginalis and fungi) ( Table 1). They also reported carrying out more therapeutic activities, such as minor surgery, etching epistaxis, use of the hyfrecator, applying pessaries and treatment of chalazion (Table 1). FP trainers spent more time directly with their patients in the surgery and on the telephone and experienced more pleasure and commitment, more job satisfaction and less job stress than non-trainers. FP trainers also reported more quality improvement activities, such as the video-recording of consultations.

### Practice level

After correction for covariates, training practices offered a significantly wider range of diagnostic and therapeutic services than non-training practices, such as audiometry, hyfrecator, spirometry, EKG, Doppler and lab service (ESR, urine sediment & culture, Haemoglobin). Diagnostic tasks were more often delegated to the practice nurse, such as spirometry and EKG as well as therapeutic tasks like Nitrogen application, compression therapy for leg ulcer, removal sutures, and wound gluing and taping sprains. Training practices also scored higher on disease management for Diabetes and CVD (Table 2). The quality system of training practices was also well developed, as there was a procedure for calibration and maintenance of equipment as well as Dutch College of GPs approved patient information, annual reports, appraisal of staff, and policy for CME of staff and other protocols for lab and treatment room. Overall, FP trainers and their practices were better organized and offer more services than non-trainers and non-training practices, although the differences are small (effect sizes are between .19 and .53 after correcting for the covariates, except for the item “ (video) recording consultation” *d* = 1.57). The differences at the practice level are somewhat larger than the FP level before and after correcting for covariates.

## Discussion

Our findings show that FP trainers offer more services and work in better organized practices than non-trainers, but the differences were small for FP trainers and small-moderate for training practices. FP trainers reported providing a wider range of services, including chronic care management, delegating more tasks and having better quality management. FP trainers enjoyed their job more, had more commitment, more job satisfaction and less job stress than their non-trainer counterparts, in spite of having more patients listed at their practice and providing a wider range of services. Overall, our findings provide evidence of the benefits and improved outcomes of FP trainers and training practices. The benchmarks set by the FP-trainers and training practices reported here could be used as the basis for Continuous Quality Improvement in the profession within both training and non-training practices.

### Explanation and comparison with other studies

The results of our study are in line with the few previous studies on some of the differences between FPs and practices in training and non-training settings [1-4]. It confirms that FP trainers and their practices are better equipped, offer more services and more quality assurance than non-trainers and their practices, and they offer more chronic disease programs and prevention. However, ours is the first study to examine such differences in detail and across so many items of service delivery/quality of care. Moreover, our findings show that FP trainers report experiencing less job stress than non-trainers, even though they report having more listed patients to whom they offer more services (than non-trainers). A possible explanation is that FPs with less job stress are more interested in becoming a trainer or that being a trainer has a stimulating and positive effect on morale and work satisfaction. It is quite likely that the presence of a trainee, while necessitating training related activities, relieves the trainers of some of their workload.

### Implications for education, policy and research

Professionally each FP and even more so each trainer needs to be informed about the quality of care of their practice. Having multiple sources of information helps facilitate comprehensive assessment, and a valid and acceptable accreditation visit method, as used in the Dutch national accreditation program, helps to provide reliable information, follow up over time and background data for all sorts of research. The FP training institutes in the Netherlands are now asking all training practices to participate in the accreditation program and variation between FP trainers on patient experiences and clinical performance can be studied using such data. It also fits into the concern of the FP training institutes to warrant that trainees get the necessary diagnostic and therapeutic skills and see the right patient mix. Giving trainers feedback on the gap in what can be learned in their practice compared to other training practices would benefit the quality of the training [17]. However, FP trainers and training practices also need to look beyond accreditation to provide a high-standard of training and use additional methods to assess the quality of the training and its impact [13,14]. More comparisons between training and non training practices and between FP trainers and non-trainers need to be made across a spectrum of other quality assessments such as pay-for-performance [18] or patient evaluations [19,20] or continuous professional development.

### Limitations

The sample included and compared FPs and practices that were all equally motivated to participate in the Dutch practice accreditation program, reducing possible bias in comparing the two groups. The analyses included a large number of variables and almost half of the differences found were significant and a third of those remained significant after correction for covariates. However, the results show comprehensively that FP trainers and their practices are better organized than non-trainers and their practices. Another limitation of the study was that we only looked at structure and processes, clinical patient outcomes were not included. We hope to present these results, when data on the treatment of chronic diseases become are available from the Dutch FP accreditation program.

## Conclusion and future directions

Our findings show that FP trainers and their host practices offer a wider range of services with more teamwork, more quality management, and the trainers report experiencing less job stress than non-trainer FPs. Moreover, hosting a FP trainer in a practice may have positive spin offs on and for other colleagues. All this may encourage more individual FPs and their host practice to become trainer and training practice respectively, as our study confirms that there are intrinsic benefits to FPs and practices to training status. However, for training and non-training settings alike, multiple quality assessments within continuous quality systems such as ongoing accreditation, provide detailed feedback on practice organization and care and highlights quality deficits that need to be addressed. There is a need for multiple sources of data showing variation between FPs and practices that offers an extra opportunity for quality improvement for those undertaking the training of FP trainees.

## Appendix 1*. The Dutch FP accreditation program*

Since 2005, FPs had the opportunity to voluntarily participate in the Dutch FP accreditation program. They receive information about the accreditation program including a questionnaire on expectations. Preparing for the first visit may take about one year.FPs gather data about their practice management and patient care followed by a pre-audit of a trained observer. Comparison with benchmarks of other FPs and practices helps to identify substandard performance stimulating FPs to make improvement plans.The first audit is carried out after delivering these plans to confirm adequate participation and to grant accreditation. It is the start of a three-year accreditation program and an assessor does the follow-up of the plans. The prolongation of the accreditation depends on having met the objectives of the improvement plans.The measurement in the accreditation program uses previously validated instruments such as VIP [11] , clinical performance [12] , and Europep [13]. The measures have been based on questionnaires for FPs and practice nurses, on structured observation by trained observers and on patient questionnaires as well as on patient data from electronic medical records.

## Competing interests

The authors declare that they have no competing interests.

## Authors' contribution

PvdH, SS-S, the main investigators analyzed the questionnaires and drafted this manuscript. JB, the project leader, was involved in all aspects of the study. AK, BB and SC participated in discussions about the reporting. All authors read and approved the final version of the manuscript.

## Pre-publication history

The pre-publication history for this paper can be accessed here:

http://www.biomedcentral.com/1471-2296/14/23/prepub
